# Concerning consequences of blocking Notch signaling in satellite muscle stem cells

**DOI:** 10.3389/fcell.2015.00011

**Published:** 2015-02-18

**Authors:** Hatem O. Kaseb, Susanne M. Gollin

**Affiliations:** ^1^Department of Human Genetics, University of Pittsburgh Graduate School of Public HealthPittsburgh, PA, USA; ^2^Department of Clinical Pathology, National Cancer Institute, Cairo UniversityCairo, Egypt

**Keywords:** stem cell, toxicity, tumor cells, Notch signaling, targeted therapy

Stem cell (SC) research holds the promise of controlling and/or curing a variety of diseases. SC applications include cell therapy, disease modeling, and developmental biology. This commentary focuses on an important study that examined Notch signaling in murine satellite muscle SCs (Lin et al., 2013). The results highlight the possible serious side effects of NOTCH inhibitors, a class of targeted drugs that are being examined for treating a variety of cancers (Krop et al., 2012). Skeletal muscles, unlike many other tissues, possess a unique regenerative capacity (Shi and Garry, 2006). Many researchers believe that this unique feature may be the key to treating myopathies, other degenerative diseases, and cancer (Jackson et al., 2010). Much of the regenerative potential of muscle depends on a population of SCs called satellite cells (Le Grand and Rudnicki, 2007).

The maintenance of stemness in adult SC is regulated by two main factors: the “SC niche” and the stemness regulatory pathways. Stemness regulatory pathways regulate stemness via SC genes and regulatory transcription factors, including the NOTCH pathway proteins. Changes in expression of these transcription factors regulate the balance between self-renewal and differentiation, vital for tissue regeneration. NOTCH is a crucial signaling pathway that regulates stemness during both embryogenesis and adulthood (Le Grand and Rudnicki, 2007). In humans, the NOTCH signaling pathway involves four receptors and five ligands, is extremely complex, and is controlled by interconnected positive and negative regulators (Fouillade et al., 2012).

To add further complexity, NOTCH family members have been shown to carry out different and sometimes opposing functions in the same tissue and/or cell type (Yin et al., 2010). The role of the Notch pathway was first identified in satellite cells from murine muscle, which were shown to have reduced regenerative potential (Le Grand and Rudnicki, 2007). Satellite cells rely on the NOTCH pathway for maintenance of quiescence (Mourikis et al., 2012), which is characterized by reversible mitotic arrest and reduced metabolic activity (Le Grand and Rudnicki, 2007; Bjornson et al., 2012). Inactivation of the NOTCH pathway is crucial for the process of differentiation (Bjornson et al., 2012). To understand the role of the Notch protein in regulating SC quiescence and muscle regeneration, Lin et al. (2013) developed a unique conditional knock-out mouse model (*Pax7-Cre+/dnMaml1+* mutant mice) in which Notch signaling is specifically blocked in the satellite SC using the dominant negative pan-Notch inhibitor, dnMaml1. The *Pax7-Cre+/dnMaml1+* mutant mice showed myopathy manifested as muscle damage and weakness as early as 6 months of age and a shortened life span of about 8 months. The satellite SC pool was totally depleted in the mutant mice due to skewing of the balance between self-renewal and differentiation to differentiation rather than self-renewal. Further, the progeny of myoblasts showed reduced proliferation compared to controls. These experiments and others post cardiotoxin (CTX)-induced injury provided evidence that Notch pathway blockade leads to loss of quiescence and depletion of the satellite SC pool. These results are concordant with reports of other studies that blocked murine Notch pathway signaling by selectively inhibiting the recombining binding protein-Jj (RBP-Jj), a nuclear factor involved in the canonical Notch signaling pathway (Bjornson et al., 2012; Mourikis et al., 2012).

In addition to its important role in muscle development, regeneration and repair, the NOTCH pathway is dysfunctional commonly in several developmental, vascular and cardiac diseases, including leukoencephalopathy and pulmonary arterial hypertension (Fouillade et al., 2012; Groth and Fortini, 2012). NOTCH is also dysregulated in a variety of human tumors, including cancers of the breast, lung, pancreas, colon, and brain (Groth and Fortini, 2012), and in head and neck squamous cell cancer (HNSCC) (Stransky et al., 2011). *NOTCH* gene mutations have been found in approximately 15% of HNSCC (Gaykalova et al., 2014), and are usually associated with loss-of-function (Stransky et al., 2011), gain-of-function, or both (Gaykalova et al., 2014). Functional studies suggest that *NOTCH1* may act as a tumor suppressor gene in HNSCC (Pickering et al., 2013).

The Lin et al. (2013) paper is pertinent to the clinical application of NOTCH inhibitors, which primarily target the presenilin/gamma-secretase aspartyl protease cleavage that releases the NOTCH intracellular domain (NICD) from the membrane (Fouillade et al., 2012). Marked toxicity affecting the gastrointestinal tract and immune systems has been demonstrated in clinical and preclinical trials, but muscle side effects have not yet been noted (Groth and Fortini, 2012). However, in combination with satellite SC exhaustion associated with aging, the side effects of NOTCH inhibitors in older cancer patients might be substantial (Carlson et al., 2009). Muscle side effects were not demonstrated in clinical trials most likely due to early termination of most trials to date (e.g., NCT01088763), in which acute side effects affected rapidly proliferating cellular compartments. Further, the role of the NOTCH pathway in regulating squamous epithelial differentiation is consistent with reports of cutaneous disorders and malignancies as side effects of NOTCH inhibitors in an Alzheimer's disease clinical trial (NCT00762411) and in *Notch1* knockout mice (Nicolas et al., 2003). These results clearly show that more research concerning the effects and side effects of inhibiting the NOTCH pathway in normal and tumor cells is crucial before targeted therapeutic approaches against the NOTCH pathway are implemented further. In addition, to avoid possibly severe and life-threatening skin or muscle-related side effects, alternative therapeutic approaches should be examined, including intra-tumoral administration of Notch inhibitors in preclinical models, as suggested by Pickering et al. (2013). Another approach may be to develop alternative small molecule inhibitors or antibodies to inhibit specific NOTCH receptors, their activating ligands, or other components of the NOTCH pathway in tumor cells (Fouillade et al., 2012). For example, Wu et al. generated antibodies that specifically target murine and human Notch receptors; short-term administration (2–3 weeks) showed fewer and less severe side effects compared to other Notch inhibitors (Wu et al., 2010).

Targeted therapies against stemness regulatory pathways like NOTCH are promising in a variety of disorders. Owing to the high possibility of muscle side effects after long-term administration, we recommend following patients by physical exam for muscle wasting and avoiding concomitant use of steroids, which can lead to myopathies. The study by Lin and colleagues (2013) highlights the critical gap in SC targeting, which is a better understanding of SCs and their complex regulatory pathways. A detailed understanding of regulatory mechanisms in SCs is essential before we can attempt to manipulate them in beneficial ways to eradicate disorders of regeneration like muscular dystrophy, and SC-driven resistance to cancer therapies, while avoiding dysregulation of normal SC processes and cellular proliferation.

## Conflict of interest statement

The authors declare that the research was conducted in the absence of any commercial or financial relationships that could be construed as a potential conflict of interest.

## Abbreviations

CTX: cardiotoxin
HGF: Hepatocyte growth factor
NICD: NOTCH intracellular domain
SC: Stem cell.
